# The impact of meteorological conditions on the concentration of alder pollen in Sosnowiec (Poland) in the years 1997–2017

**DOI:** 10.1007/s10453-018-9524-8

**Published:** 2018-06-12

**Authors:** Katarzyna Dąbrowska-Zapart, Kazimiera Chłopek, Tadeusz Niedźwiedź

**Affiliations:** 1 Department of Paleontology and Stratigraphy, Faculty of Earth Sciences, University of Silesia, Będzińska 60, 41-200 Sosnowiec, Poland; 2 Department of Climatology, Faculty of Earth Sciences, University of Silesia, Będzińska 60, 41-200 Sosnowiec, Poland

**Keywords:** Alder pollen, *Alnus*, Weather types, Meteorological conditions, Statistical analysis

## Abstract

The aim of the work was to compare the alder pollen seasons in the years 1997–2017 in Sosnowiec. The measurements of pollen concentration were taken with the volumetric method using Burkard’s apparatus. The impact of atmospheric conditions on the daily alder pollen grain concentration, the annual totals, and the duration of pollen seasons were studied. The dependency between each meteorological condition and different features of the alder pollen season was determined by using Pearson’s correlation coefficients, variance analysis with multiple comparison tests, and the linear regression model using backward elimination. It was proven that the temperatures directly preceding the pollination, i.e. the January and February temperatures as well as those from the period from 210 to 180 days preceding the beginning of the season, have the greatest impact on the beginning of the alder pollen season. The value of the daily alder pollen concentration in Sosnowiec showed a positive statistically significant correlation with the air temperature and sunshine duration and a negative correlation with the thickness of the snow cover and air relative humidity. The daily concentration also depended on the type of the weather front, direction of air mass inflow, and the type of the inflowing air mass. The season temperatures and the thermal conditions which were present in the summer of the preceding year impacted the annual totals (SPI) of the alder pollen grains.

## Introduction

In Poland, the genus *Alnus* is represented by three species. Two of them are trees, *Alnus* glutinosa and *Alnus incana*, and the third one (*Alnus viridis*) is a small shrub the occurrence of which is limited only to the Bieszczady Mountains. In the city of Sosnowiec and adjacent areas, two alder species were found: *Alnus glutinosa* and *Alnus incana* (Zając and Zając 2001).

In northern and central Europe, the *Alnus* pollen is one of the main causes of pollen allergy (Rapiejko et al. 2004; d’Amato et al. 2007; Jantunen et al. 2012). Alder pollen concentrations are high, and they often exceed the threshold values causing allergy symptoms and they also come into cross-reactions with hazel, birch, hornbeam, oak, and beech pollen (Matthiesen et al. 1991; Neudecker et al. 2001; Weryszko-Chmielewska and Rapiejko 2007; Kaszewski et al. 2008). The threshold alder pollen concentration, wherein in persons suffering from hypersensitivity allergic symptoms are observed, amounts to 45 grains/m^3^ of air in Poland, whereas at a concentration of 80 grains/m^3^ of air, pollinosis is present in all people allergic to alder pollen (Rapiejko et al. 2007).

The presence of *Alnus* pollen grains in the air is dependent on the course of the plants’ blossoming. The conducted pollen monitoring shows significant time differences in the appearance of pollen, quantity, and the length of the pollen season. The weather conditions, particularly air temperature, impact the times of pollen presence in the air (Frenguelli et al. 1991; Emberlin et al. 2002; Kasprzyk et al. 2004; Emberlin et al. 2007). Many years of research on the dynamics of pollen seasons in the conditions of Poland (Sosnowiec) have shown high variability of *Alnus* seasons.

**Fig. 1:**
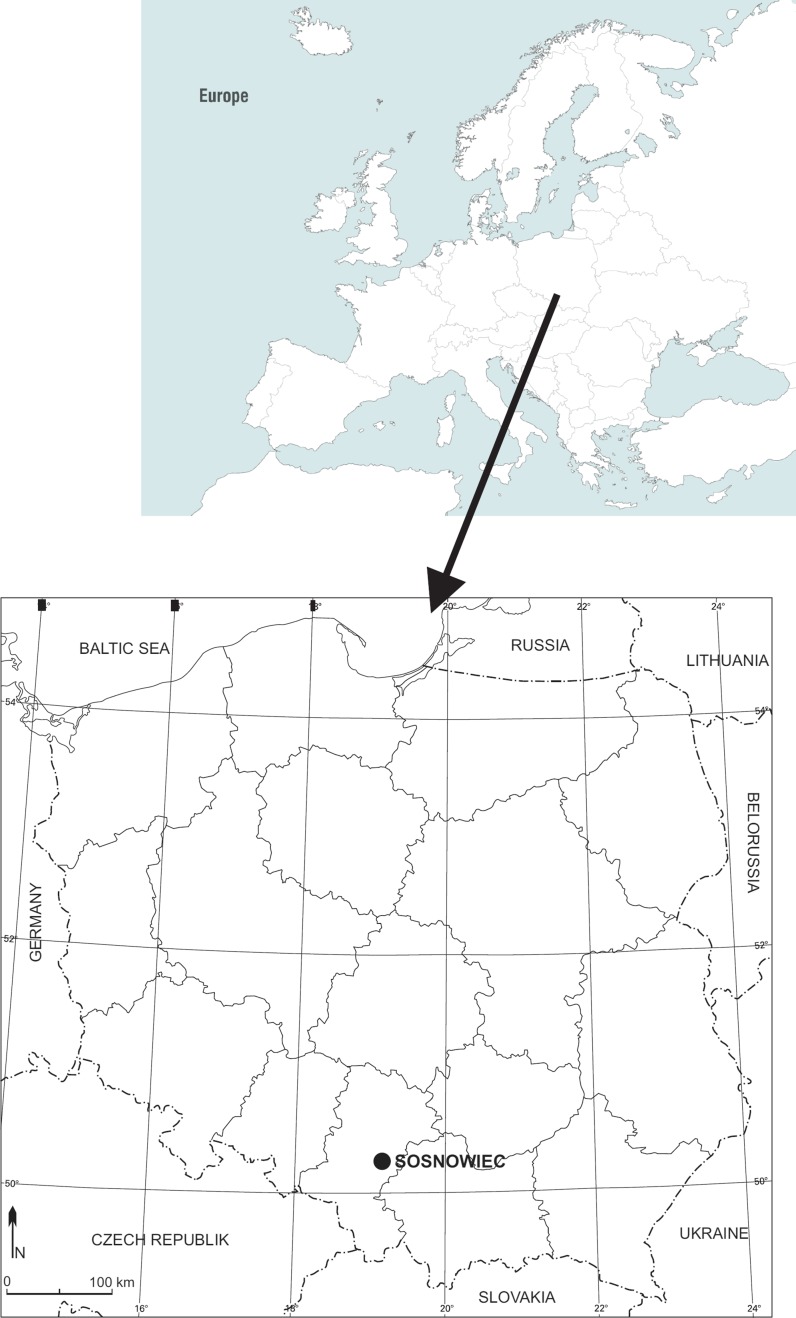
Geographical location of Sosnowiec in Central Europe. Reproduced with permission from www.vecteezy.com/map-vector

Sosnowiec is located in southern Poland in the eastern part of the Silesian Upland (Fig. 1). Despite a significant concentration of residential and industrial buildings, Sosnowiec is an area where different habitats are overgrown with a significant number of vascular plant species, which belong to many botanic families. According to Jędrzejko (1993), the total area occupied by green areas in the city’s territory constitutes approximately 24.7% of the area.

In the territory of the Silesian Upland, where Sosnowiec is located, the influences of different air masses intersect and therefore the climate is characterised by significant variability and irregularity of the course of climatic elements. Sosnowiec lies in the temperate climate zone—intermediate between the oceanic and continental ones. The weather for most of the days in a year (65%) is shaped under the influence of maritime polar air inflowing from above the Atlantic Ocean. The average annual temperature is at 9.2 °C; the warmest month is July (19.5 °C), while the coldest one is January (− 1.2 °C). The average total annual precipitation is 735 mm. Snowfall occurs for approximately 50 days a year. The average number of days with snow cover is 66; the average snow cover thickness amounts to 25 cm. The high weather variability and the occurrence of precipitation accompany weather fronts which move above the territory of Sosnowiec 40.5% of days per year. Among the winds present in Sosnowiec, the dominating wind comes from the western direction, followed by the southern, northern–western, and southern–western directions. The average wind speed in Sosnowiec is estimated at approximately 3.1 m s^−1^ (Niedźwiedź and Małarzewski 2016).

The aim of the study was to compare the concentrations of alder pollen grains during 21 years of research and to investigate the impact of meteorological conditions on different characteristics of pollen seasons of this taxon in Sosnowiec.

## Materials and methods

Alder pollen concentrations were analysed based on the data obtained in Sosnowiec in the years 1997–2017. The aerobiological measurements were taken using the volumetric method with the use of a Burkard-type apparatus. The apparatus was located at a height of 20 m in the area of the buildings belonging to the Faculty of Earth Sciences of the University of Silesia in Sosnowiec, in the northern part of Pogoń, a Sosnowiec district, which is characterised by a residential block low-density housing. The geographical coordinates of the measurement point are: 50°17′50″N and 19°08′20″E. Nearby, at a height of 263 m a.s.l. a weather station of the Department of Climatology is located, the weather data of which were used. Some data from the synoptic station of the Institute of Meteorology and Water Management in Katowice, located approximately 10 km to the south-west of Sosnowiec, were also used. The average daily and monthly values of meteorological elements, such as average, ground, maximum, and minimum air temperature and the daily temperature amplitude, sunshine duration, relative humidity, and water vapour pressure, average and maximum wind speed, precipitation, presence and thickness of snow cover, direction and type of atmospheric circulation, were used in the research (Niedźwiedź 1981, 2004).

The pollen grain analysis was carried out using a light microscope after the specimens were stained using alkaline fuchsin on the surface of four horizontal stripes (Mandrioli et al. 1998). The duration of pollen seasons was determined using the 98% method, assuming for its beginning and end days when 1 and 99% of the total annual pollen grains appeared, respectively (Emberlin et al. 1994; Spieksma and Nikkels 1998).

In the season’s characteristics, the following parameters were determined: the beginning and end dates, duration, the total number of pollen grains, maximum daily concentration, date of occurrence of the highest concentration, and the number of days in which concentration exceeded the threshold values (45 and 80 grains/m^3^) (Rapiejko et al. 2007).

In order to analyse the differences in the course of the pollen seasons in the years under study, descriptive statistics such as arithmetic mean, minimum and maximum number of pollen grains, standard deviation, and coefficient of variation were used. For each pollen season feature, a variation was determined by using linear regression. In order to analyse each variable using the basic measures of descriptive statistics, the daily dates of each characteristic of the pollen seasons were replaced by the number of days which had elapsed since the beginning of the year. To evaluate the coefficient of variation (*V*), the following interpretation was applied: *V* < 20%—low variation, 20% < *V* < 40%—average variation, 40% < *V* < 100%—high variation.

In order to check whether the distribution of variables is close to the normal distribution, Kolmogorov–Smirnov and Shapiro–Wilk tests were applied. Due to the fact that the distribution of variables was close to normal and referring to the central limit theorem (the law of large numbers), which states that as the number of observations increases, the distribution of variables approaches the normal distribution, it was decided to apply the appropriate parametric tests. A statistical error risk was estimated at the significance level: *α* = 0.05, 0.01 and 0.001 using the Statistica version 9.

Pearson’s r correlation analysis and variance analysis with LSD post hoc multiple comparison tests were carried out and a linear regression model was built, using backward elimination, to determine the influence of the weather conditions on the studied features of alder pollen seasons. The correlation strength was measured using the following ranges: 0–0.3 low correlation, 0.3–0.5 moderate, 0.5–0.7 strong, 0.7–1 very strong correlation. In order to verify whether the analysed weather factors are related to some extent, a factor analysis was carried out using the method of the principal component analysis.

## Results

In the period of 21 years of research, significant differences in the studied characteristics of alder pollen seasons were observed. Definitely, the least varied were the dates of the end of the pollen season and the dates of the maximum pollen grain concentration during the day, which confirms the low coefficient of variation (Table 2). The characteristics of the pollen seasons which turned out to be the most variable in the studied years were: maximum value of daily concentration, value of the annual total, and the beginning date of the season (Table 2). The annual totals ranged from 429 grains in 2009 to 4742 grains in 2003 (Table 1). The analysis of the annual totals of alder pollen grain in Sosnowiec did not demonstrate any existing cyclicality in the occurrence of years with high values of annual totals alternately with years of low pollen production. In the series under study, there are years with lower values of pollen grains separated by years with a high annual total, but not in regular time intervals.

**Table 1:** Characteristics of alder pollen seasons in Sosnowiec

Year	Beginning of the pollen season	End of the pollen season	Duration of the pollen season	Maximum concentration	Date of max concentration	Annual total	**≥** 45 grains/m^3^	**≥** 80 grains/m^3^
1997	21.02 (52)	15.04 (105)	53	109	2.03 (61)	533	4	2
1998	18.01 (18)	23.03 (82)	64	211	22.02 (53)	1817	12	8
1999	5.02 (36)	14.04 (104)	68	476	3.03 (62)	2302	10	5
2000	4.02 (35)	2.05 (123)	88	85	29.02 (60)	823	5	1
2001	9.02 (40)	4.04 (94)	54	1172	12.03 (71)	4707	12	9
2002	1.02 (32)	2.05 (122)	90	76	19.02 (50)	931	6	0
2003	11.03 (70)	20.04 (110)	40	1213	27.03 (86)	4742	15	12
2004	6.02 (37)	12.04 (103)	66	996	18.03 (78)	3270	9	5
2005	11.03 (70)	20.04 (110)	40	324	27.03 (86)	1405	9	6
2006	21.03 (80)	16.05 (136)	56	361	1.04 (91)	2104	12	7
2007	16.01 (16)	29.04 (119)	103	92	12.03 (71)	822	6	2
2008	26.01 (26)	21.03 (81)	55	819	24.02 (55)	4691	16	9
2009	5.03 (64)	4.05 (124)	60	70	17.03 (76)	429	2	0
2010	2.03 (61)	10.04 (100)	39	757	21.03 (80)	3580	9	9
2011	6.03 (65)	15.04 (105)	40	195	22.03 (81)	781	5	2
2012	2.03 (62)	3.04 (94)	32	280	19.03 (79)	1129	5	4
2013	5.03 (64)	26.04 (116)	52	623	12.04 (102)	2060	7	6
2014	1.02 (32)	17.03 (76)	44	337	9.03 (68)	3136	18	15
2015	21.02 (52)	8.04 (98)	46	163	8.03 (67)	1217	9	4
2016	4.02 (35)	8.04 (99)	64	715	22.02 (53)	3447	14	9
2017	24.02 (55)	26.03 (85)	30	266	5.03 (64)	1407	9	6

The numbers in parentheses indicate the consecutive day from the beginning of the pollen season

In 2003, the highest daily concentration, which reached 1213 grains/m^3^, was also found. The beginning of the season fell in mid-February on average. Alder pollen grain appeared in Sosnowiec air in 2007 at the earliest, already on the 16th of January, with the latest in 2006—on the 21st of March, which means more than 2 months later. In the years when the pollen season started late (2003, 2005, 2006, 2011, and 2013), a delay in the date of occurrence of the seasonal maximum was also observed (Table 1). The duration of the pollen seasons was 56 days on average. The longest lasting season was determined in 2007 (103 days) and the shortest one in 2017 (30 days). We may qualify the variation of this characteristic (34%) to average variation, as it fits in the range of 20% < *V* < 40% (Table 2).

**Table 2:** Statistics of alder pollen season in Sosnowiec

Data from the years 1997–2017						
	Beginning of the season (days)	End of the season (days)	Duration of the season (days)	Maximum daily concentration (grains/m^3^)	Maximum concentration date	Annual total
$\overline{x}$	48	104	56	445	71	2160
Min	16 (2007)	76 (2014)	30 (2017)	70 (2009)	50 (2002)	429 (2009)
Max	80 (2006)	136 (2006)	103 (2007)	1213 (2003)	102 (2103)	4742 (2003)
SD	18.5	15.8	19.2	367.3	13.9	1435.7
*V* (%)	38.7	15.2	34	82.6	19.5	66.5

$\bar{x}$, arithmetic mean; Min, minimal concentration of pollen grains; Max, maximum concentration of pollen grains; SD, standard deviation; *V*, coefficient of variation

The course of the pollen seasons in 2003 and 2005 turned out to be very similar. The pollen season in both cases started on the same day and ended on the same day. The date of the maximum concentration was also identical; however, the concentration values differed (Table 1).

It turned out that in the years 2003, 2008, 2014, and 2016 were the most days with a concentration exceeding the threshold values in the case of which symptoms in people allergic to alder pollen allergens were recorded (Table 1).

Beside each plant’s individual rhythm of pollination, meteorological elements are important factors that impact pollen air concentration conditions. Also in the case of alder, they undoubtedly determine the presence of this taxon’s pollen in the air. The conducted statistical analyses showed the weather conditions’ impact on the seasonal and daily alder pollen grain variation. The air temperature, which in the alder pollination period is subject to high fluctuation, seems to be the most important element here.

The beginning of the alder pollen season is very difficult to predict, and the dates of the season’s beginning show significant differences each year. The values of the correlation coefficient calculated between the mean of the selected meteorological characteristics from the period from 5 to 210 days preceding the season and the alder pollen season beginning date show that not only the conditions directly preceding the pollination date impact the date of the appearance of pollen grains in the air, but also those which are present a long time before the beginning of the pollen season (Table 3). The highest correlation coefficients were calculated for the average, minimum, maximum, and ground temperature from the period of 180 and 210 days preceding pollination (Table 3). High coefficient values were also obtained for temperatures from the period from 25 to 40 days. This means that the temperature from the period from the summer of the preceding year (June–August) to the day when pollination starts has the greatest impact on the dates of the alder pollen season beginning. The temperatures from the period directly preceding the dates of beginning of the alder pollen season (25–40 days) are also important. The higher the temperatures in this period are, the faster the alder pollen seasons begin. Statistically significant correlation coefficients were also determined for precipitation from the period of 15 days before the season and the relative humidity from a period from 25 to 60 days. No significant dependency was discovered between the pollen season beginning date and sunshine duration before the season (Table 3).

**Table 3:** Correlation coefficient values determining the dependency between the beginning of the pollen season and the weather conditions in the preceding period in a range from 5 to 210 days

Number of days preceding the season	Weather elements						
	Average T	Minimum T	Maximum T	T 5 cm	Precipitation	Relative humidity	Sunshine duration
5	− 0.52	− 0.52	− 0.45	− 0.49	0.00	0.08	0.06
10	− 0.54	− 0.40	− 0.52	− 0.64*	− 0.55	− 0.18	0.11
15	− 0.41	− 0.48	− 0.54	− 0.55	− 0.72**	− 0.37	0.45
20	− 0.45	− 0.60*	− 0.59	− 0.62*	− 0.55	− 0.60	0.48
25	− 0.83***	− 0.84***	− 0.70**	− 0.83***	− 0.45	− 0.76*	0.52
30	− 0.84***	− 0.84***	− 0.72**	− 0.82***	− 0.36	− 0.80***	0.61
40	− 0.80***	− 0.79**	− 0.71**	− 0.80***	− 0.41	− 0.66*	0.60
60	− 0.69*	− 0.65*	− 0.62*	− 0.62*	− 0.05	− 0.80***	0.55
90	− 0.64*	− 0.71**	− 0.65*	− 0.67*	− 0.14	− 0.53	0.31
120	− 0.70**	− 0.69*	− 0.72**	− 0.68*	0.18	− 0.08	− 0.23
150	− 0.71**	− 0.75**	− 0.78**	− 0.75**	− 0.32	− 0.03	− 0.32
180	− 0.89***	− 0.87***	− 0.88***	− 0.84***	− 0.44	0.35	− 0.31
210	− 0.91***	− 0.89***	− 0.92***	− 0.87***	− 0.70	0.22	− 0.43

*p < 0.05; **p < 0.01; ***p < 0.001

From among the average monthly values of the weather elements which impact the alder pollen season beginning dates, it turned out that the most important were the January and February temperatures (Table 4). High positive correlation coefficients signify that high temperatures in January and February accelerate the appearance of alder pollen in the air. Particularly important were the temperatures in the period of January–February.

**Table 4:** Correlation coefficients between the average monthly values of the weather conditions and the alder pollen season beginning date

Meteorological conditions	Beginning of the pollen season
Average temperature January	− 0.65*
Minimum temperature January	− 0.66*
Maximum temperature January	− 0.78**
Ground temperature January	− 0.67*
Average temperature February	− 0.79**
Minimum temperature February	− 0.81***
Maximum temperature February	− 0.74**
Ground temperature February	− 0.81***
Average temperature January–February	− 0.87***
Minimum temperature January–February	− 0.91***
Maximum temperature January–February	− 0.84***
Ground temperature January–February	− 0.86***

*p < 0.05; **p < 0.01; ***p < 0.001

The results of Pearson’s *r* correlation between the number of alder pollen grains during the day and each element which determines the weather conditions are presented in Table 5.

**Table 5:** Results of Pearson’s *r* correlation between the number of alder pollen grains and the weather conditions

Weather conditions	Alder pollen grain daily concentration
Precipitation	− 0.03
Snow cover thickness	− 0.09**
Average wind speed	0.03
Maximum wind speed	0.04
Maximum temperature	0.19***
Minimum temperature	0.11***
Ground temperature	0.09**
Average temperature February	0.17***
Temperature amplitude	0.15***
Sunshine duration	0.07*
Relative humidity	− 0.08**

*p < 0.05; **p < 0.01; ***p < 0.001

The results of the correlation analysis showed that the number of alder pollen grains was not statistically correlated with precipitation and the average and maximum wind speed. A positive correlation was found between the number of alder pollen grains and the maximum temperature *r* = 0.19; *p* < 0.001, minimum temperature *r* = 0.11; *p* < 0.001, ground temperature *r* = 0.09; *p* < 0.001, average temperature *r* = 0.15; *p* < 0.001, and sunshine duration *r* = 0.07; *p* < 0.05. The high values of these variables were connected with the higher number of alder pollen grains during the day.

However, negative correlation was determined between the number of alder pollen grains and snow cover thickness *r* = − 0.09; *p* < 0.01 and relative humidity *r* = − 0.08; *p* < 0.01. The presence of snow cover and high relative humidity of the air had an impact on the reduction in the number of alder pollen grains.

Next, an analysis of variance for the nominal variable, i.e. the weather front (Table 6), inflow direction (Table 7), and air mass type (Table 8), was carried out. This method compares the average values taking into account the grouping factor. Intra-group comparisons were made using LSD post hoc tests. The results in Table 6 were given as the mean values and standard deviations.

**Table 6:** Results of the variance analysis of the number of alder pollen grains on account of the weather front type

Front type	Frequency %	Alder pollen grain number
No front	59.2	30.06 (± 91.25)
Cold	10.5	43.74 (± 115.91)
Warm	14.0	66.36 (± 177.28)
Occluded	5.5	28.63 (± 93.27)
Stationary front	5.2	34.38 (± 83.93)
Various fronts	5.6	34.33 (± 64.41)
*p*		*p* < 0.05*

**Table 7:** Results of the analysis of variance of the alder pollen grain number on account of the air mass inflow direction

Direction	Frequency %	Alder pollen grain number
N + NEa	7.3	15.96 (± 66.58)
E + SEa	11.6	27.43 (± 58.49)
S + SWa	5.8	55.55 (± 155.46)
W + NWa	12.6	53.67 (± 147.86)
Ca + Ka	12.2	26.46 (± 79.04)
N + NEc	7.3	24.71 (± 50.74)
E + SEc	6.2	14.06 (± 47.28)
S + SWc	8.9	72.44 (± 174.67)
W + NWc	12.9	45.52 (± 111.2)
Cc + Bc	12.9	21.01 (± 57.13)
*x*	2.3	27.09 (± 45.6)
*p*		*p* < 0.001***

Anticyclonic situations: N + NEa, E + Sea, S + Swa, W + NWa, Ca + Ka; Ca, central anticyclone situation (high centre); Ka, anticyclonic wedge or ridge of high pressure

Cyclonic situations: N + NEc, E + Sec, S + SWc, W + NWc, Cc + Bc; Cc, central cyclonic, centre of low; Bc, trough of low pressure (different directions of air flow and frontal system in the axis of through)

*x*, undefined type

The letters: N, NE, E, SE, S, W, NW, SW indicate the directions of air mass inflow

**Table 8:** Results of the variance analysis of the number of alder pollen grains on account of the air masses

Air mass type	Frequency %	Alder pollen grain number
A	13.1	7.43 (± 16.36)
mP	11.6	33.8 (± 72.84)
omP	32.5	28.98 (± 79.69)
cP	20.0	37.25 (± 116.65)
wmP	11.6	92.13 (± 183.91)
T	0.7	5.1 (± 13.41)
v.a.m.	10.6	41.86 (± 125.6)
*p*		*p* < 0.001***

omP, old maritime polar air (transformed); wmP, warm maritime polar air; fmP, fresh maritime polar air; cP, continental polar air; A, arctic air; T, tropical air; v.a.m., various air masses at the fronts

**Fig. 2:**
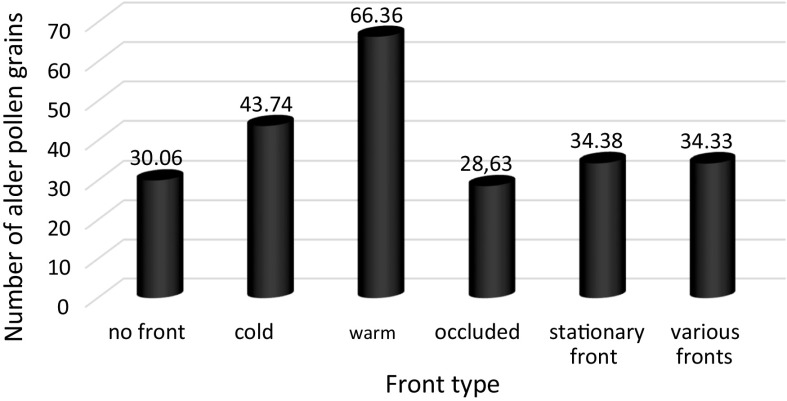
Average number of alder pollen grains broken down into weather front type

The analysis showed that there was a statistically significant dependence between the number of alder pollen grains and the weather front type *p* < 0.05 (Table 6). Multiple comparison tests showed that the highest alder pollen grain number was recorded in the case of a warmer front (*M* = 66.36; SD = 177.28) and the lowest alder pollen grain number in the case of the occluded front (*M* = 28.63; SD = 93.27) (Fig. 2).

**Fig. 3:**
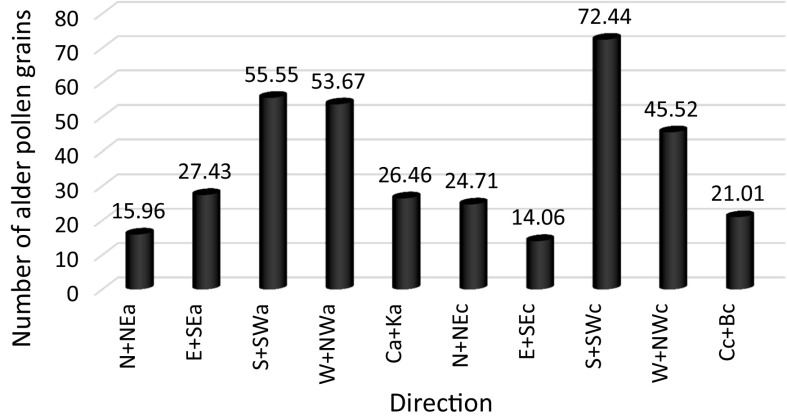
Average number of alder pollen grains broken down into the direction of air mass inflow. Anticyclonic situations: N + NEa, E + Sea, S + Swa, W + NWa, Ca + Ka; Ca, central anticyclone situation (high centre); Ka, anticyclonic wedge or ridge of high pressure; cyclonic situations: N + NEc, E + Sec, S + SWc, W + NWc, Cc + Bc; Cc, central cyclonic, centre of low; Bc, trough of low pressure (different directions of air flow and frontal system in the axis of through); *x*, undefined type. The letters: N, NE, E, SE, S, W, NW, SW indicate the directions of air mass inflow

The analysis showed that there was a statistically significant dependence between the number of alder pollen grains and the inflow of air masses *p* < 0.001 (Table 7). Multiple comparison tests showed that the highest average number of alder pollen grains was recorded in the case of an inflow of warm air from the S + SWc (cyclonic situation from the south and south-west) direction (*M* = 72.44; SD = 174.67) and the lowest number of alder grains was recorded for the E + SEc (cyclonic situation from the east and south-east) direction (*M* = 14.06; SD = 47.28) and N + NEa (anticyclonic situation from the north and north-east) direction (*M* = 15.96; SD = 66.58), from which the cold polar continental air usually flows (Fig. 3).

**Fig. 4:**
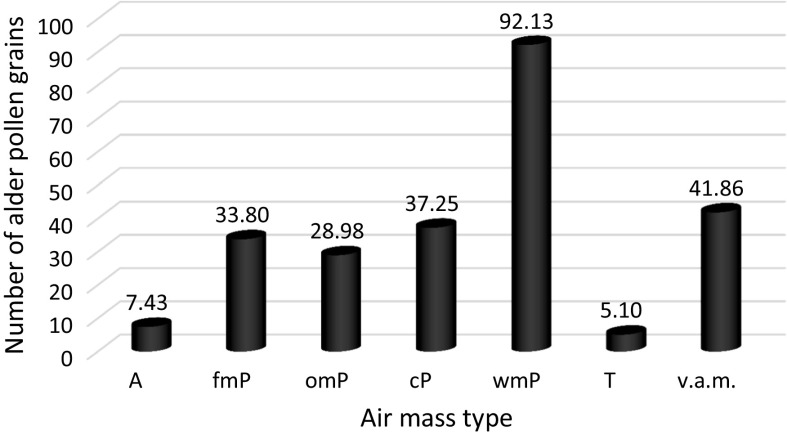
Average number of alder pollen grains broken down into air masses. omP, old maritime polar air (transformed); wmP, warm maritime polar air; fmP, fresh maritime polar air; cP, continental polar air; A, arctic air; T, tropical air; v.a.m., various air masses at the fronts

The analysis showed that there was a statistically significant dependence between the number of alder pollen grains and the type of inflowing air masses *p* < 0.001 (Table 8). The multiple comparison tests showed that the highest number of alder pollen grains was recorded in the case of inflow of warm maritime polar air wmP (*M* = 92.13; SD = 183.91) and the lowest number of alder grains was recorded for tropical air T (*M* = 5.1; SD = 13.41), which is very seldom present during pollination (0.7%), and arctic air A (*M* = 7.43; SD = 16.36), whose frequency in the period between February and April was equal to 13.1% (Fig. 4).

In order to study the impact of the weather conditions on the daily concentration of alder pollen grains, a linear regression model was built using backward elimination (Table 9). This method enables maintaining the assumption of the multiple regression analysis, stating no correlation between the predictors. Those variables which are not strongly correlated and describe the studied phenomenon in a universal way are maintained in the model.

**Table 9:** Results of the regression analysis for the impact of the weather conditions on the number of alder pollen grains

	*df*	*F*	*p*	*R* ^2^
Regression	5	13.51	0.000	0.05
Residual	1146			

*df*, number of degrees of freedom; *F*, ANOVA statistics; *p*, statistical significance; *R*^2^, determination coefficient

The model of the meteorological variables’ impact on the alder pollen number turned out to be statistically significant *F* (5.1146) = 13.51; *p* < 0.001; *R*^2^ = 0.05. The variation of the weather conditions explained in 5% the variation of the alder pollen grain number during the day (Table 9).

It was discovered that the average wind speed *t* = 1.71; *p* = 0.087, average temperature *t* = 4.72; *p* < 0.001, temperature amplitude *t* = 4.72; *p* < 0.001, and sunshine duration *t* = − 1.84; *p* = 0.066 (Table 10) were statistically significant predictors (or at the border of a statistical tendency). It turned out that the strongest predictors were the average temperature *β* = 0.66 and the relative humidity *β* = − 0.58. The average temperature increase by one unit (1 °C) caused an increase in the alder pollen number by *B* = 14.36, whereas an increase in the relative humidity by one unit (1%) caused a decrease in the number of the alder pollen grain number by *B* = − 18.66 (Table 10).

**Table 10:** Results of the regression analysis for the impact of the weather conditions on the number of alder pollen grains

	Unstandardised coefficients	Standardised coefficients		*t*	*p*
	*B*	Standard error	Beta		
(Constant)	88.53	28.78		3.08	0.002
Average wind speed	5.00	2.91	0.05	1.72	0.087
Average temperature	14.36	3.04	0.66	4.72	0.000
Temperature amplitude	5.62	1.19	0.24	4.72	0.000
Sunshine duration	− 2.25	1.22	− 0.09	− 1.84	0.066
Relative humidity	− 18.66	4.59	− 0.58	− 4.07	0.000

*B*, unstandardised impact slope; Beta, standardised impact slope; *t*, Student’s *t* statistics; *p* statistical significance

The results of the correlation analysis showed that the total number of alder pollen grains in the season (SPI) depended not only on the season’s weather conditions, but also on those which occurred in the year preceding pollination. The number of alder pollen grains was statistically significant and negatively correlated with the maximum temperature *r* = − 0.45; *p* < 0.05, minimum temperature *r* = − 0.44; *p* < 0.05, average temperature *r* = − 0.46; *p* < 0.05, and precipitation *r* = − 0.42; *p* < 0.05 in the season. The high levels of these variables in the years under study were connected with a lower number of alder pollen grains in the pollination period (Table 11).

**Table 11:** Results of Pearson’s *r* correlation between the annual total number of alder pollen grains and the weather conditions for annual data

Weather conditions	Alder pollen grain number
Precipitation	− 0.42*
Snow cover thickness	0.15
Average wind speed	− 0.28
Maximum wind speed	− 0.12
Maximum temperature	− 0.45*
Minimum temperature	− 0.44*
Ground temperature	− 0.32
Average temperature	− 0.46*
Temperature amplitude	− 0.32
Sunshine duration	− 0.40
Humidity	0.37

*p < 0.05

From among the elements impacting the annual totals in the year preceding pollination, the highest correlation coefficients were obtained for the temperatures from the period from July to September (Table 12). This means that the annual totals of alder pollen grains may impact the average temperatures from the summer of the preceding year. A statistically significant correlation was also discovered in the case of sunshine duration from the period of July–September of the year preceding pollination *r* = 0.44; *p* < 0.05. On the other hand, a negative correlation was discovered in the case of precipitation from the period of July–September *r* = − 0.46; *p* < 0.05 (Table 12).

**Table 12:** Correlation coefficients between meteorological elements in the year preceding pollination and the annual total of alder pollen grains

Weather conditions	Alder pollen grain number
Precipitation July–September	− 0.46*
Average wind speed	− 0.20
Maximum wind speed July–September	− 0.24
Maximum temperature July–September	0.65**
Minimum temperature July–September	0.66**
Ground temperature July–September	0.64**
Average temperature July–September	0.73**
Temperature amplitude July–September	0.55**
Sunshine duration July–September	0.44*
Humidity	0.37

*p < 0.05; **p < 0.01

The duration of the alder pollen seasons was positively correlated with the relative humidity *r* = 0.47; *p* < 0.05 and the average *r* = 0.56; *p* < 0.01 and maximum *r* = 0.59; *p* < 0.01 wind speed in the season, while it was negatively correlated with the average *r* = − 0.46; *p* < 0.05, minimum *r* = − 0.44; *p* < 0.05, maximum *r* = − 0.45; *p* < 0.05, and ground temperature in the season *r* = − 0.47; *p* < 0.05 (Table 13), whereas no significant dependence was found between the duration of the alder pollen seasons and the directions of air inflow and the air mass and weather front types.

**Table 13:** Results of Pearson’s *r* correlation between the pollen season duration and the weather conditions

Weather conditions	Duration of the pollen season
Precipitation	− 0.19
Snow cover thickness	− 0.28
Average wind speed	0.56**
Maximum wind speed	0.59**
Maximum temperature	− 0.45*
Minimum temperature	− 0.44*
Ground temperature	− 0.47*
Average temperature	− 0.46*
Temperature amplitude	− 0.32
Sunshine duration	− 0.40
Relative humidity	0.47*

*p < 0.05; **p < 0.01

## Discussion

**Fig. 5:**
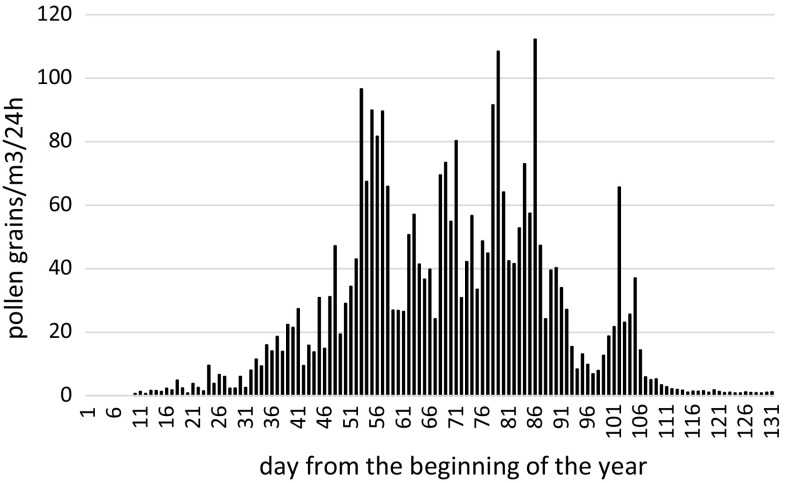
The average course of pollen seasons from the years 1997–2017

Pollen grain concentration monitoring is an important tool used for evaluating the degree of exposure to allergens, diagnostics in the case of allergy occurrence, evaluation of treatment efficiency, or pollen allergy prophylaxis. Pollen seasons are characterised by a high variation, both on the account of duration and pollen grain concentration (Spieksma et al. 2003). Both weather conditions during blossoming and flower bud formation and the genetic conditions of the plants connected with their productivity influence the course of the pollen seasons. Thus, the longer the series of measurement is, the more the complete data we may obtain on the pollen season dynamics of each taxon. The *Alnus* pollen season in Sosnowiec started on average on the 48th day counted from the beginning of the year and ended on the 104th day (Table 2). The duration of the pollen season equalled 56 days. The lowest variation in the years under study was shown in the dates from the season’s end and the dates of occurrence of the maximum daily pollen concentration, which has also been found in other Polish cities (Wołek and Myszkowska 2008; Myszkowska et al. 2010, Kasprzyk 2011). The highest coefficient of variation was characteristic for the highest daily concentrations and the annual totals of pollen grains. The average course of pollen seasons from the years 1997–2017 is shown in Fig. 5.

In the case of most anemophilous trees, bursting of the thecae is mainly controlled by the temperature, humidity, and wind speed (Pacini and Hesse 2004). In particular, the pollination seasons of trees which blossom at the turn of winter and spring, including the *Alnus*, are regulated by the dynamically changing weather conditions during that time. Research conducted in many European countries shows that the beginning and duration of the *Alnus* pollen seasons have been changing year-on-year (Dahl and Strandhede 1996; Weryszko-Chmielewska et al. 2001; Rodriguez-Rajo et al. 2006; Ranta and Satri 2007; Kalinovych et al. 2016). The *Alnus* pollen season beginning dates may vary in subsequent years from 2 weeks and up as much as 1.5 months (Piotrowicz and Myszkowska 2006; Emberlin et al. 2007; Smith et al. 2007; Stach et al. 2007; Myszkowska et al. 2010, Piotrowska-Weryszko 2013). In Sosnowiec, the difference between the earliest (16 January 2007) and the latest appearance of *Alnus* pollen (21 March 2006) equalled more than 2 months (Table 1). This was caused by the thermal conditions which preceded the pollen season. It was demonstrated that high temperatures in the months preceding pollination (January–February) accelerate the beginning of the seasons of the taxon studied. In 2007, January was characterised by the highest average temperature in the years studied (3.6 °C), which undoubtedly influenced the very early appearance of alder pollen in the air. In 2006, when the latest beginning of the alder pollen season was observed, the average January temperature was − 7.2 and − 2.7 °C in the case of February. A late beginning of the alder pollen seasons was also observed in the years 2003 and 2005, which was caused by low January, February, and March temperatures. Similar dependence for this taxon was stated in Lublin by Piotrowska-Weryszko (2013).

When forecasting the start of tree pollen seasons, sometimes the so-called cumulative temperature is used (Garcia-Mozo et al. 2000; González-Parrado et al. 2006; Linkosalo et al. 2017). This method assumes that anthers only open after absorbing a specific dose of thermal energy, which varies for different tree taxa. It is based on creating cumulative series most often from January 1 of a given year as the sum of the average daily temperature values exceeding certain thresholds until the day, which is marked as the beginning of the pollen season.

Due to the fact that the air temperature undoubtedly has the greatest influence on the appearance of alder pollen grains in the air, cumulative temperatures were determined from January 1 till the beginning of the season. Two temperature thresholds were used: above 0 °C and above 5.5 °C. The alder pollen was present in the air when the sum of average temperatures higher than 0 °C ranged from 19.5 to 91.2 °C, and above 5.5 °C even from 0 °C to 69.3 °C. The sums of temperatures contained in such large ranges do not allow for unambiguous prediction of the alder pollen season start in Sosnowiec.

The analyses demonstrated that not only the weather conditions directly preceding the pollination date impact the beginning of the pollen season, but also those which are present a long time before the beginning of the pollen season (Table 3). It is worth underlining that the highest correlation coefficients were obtained for the averages from 180 and 210 days, which means that the temperatures from the period of the preceding summer (June–August) until the day in which pollination occurs are of the greatest importance. The higher the average temperature from this period is, the faster the alder starts pollinating. The temperatures from the period directly preceding the dates of beginning of the alder pollen season (25–40 days) are also important. Numerous authors state that the temperature in the period preceding pollination has a significant impact on the beginning of the pollen seasons of the trees which start their pollination at the turn of spring and winter (Frenguelli et al. 1991; Frei 1998; Rodríguez-Rajo et al. 2004; Emberlin et al. 2007; Kim et al. 2011; Piotrowska-Weryszko 2013). Statistically significant correlation coefficients were also determined for precipitation from the period of 15 days before the season and the relative humidity from a period from 25 to 60 days.

The statistical analysis of the dependence between the daily alder pollen concentration and the weather conditions confirmed the results also obtained by other authors (Jato et al. 2002; Rodríguez-Rajo et al. 2003; Puc 2007; Myszkowska et al. 2007; González Parrado et al. 2009; Puc and Kasprzyk 2013; Dahl et al. 2013; Stępalska et al. 2016; Borycka and Kapsrzyk 2018; Bruffaerts et al. 2018). Statistically significant, positive correlation coefficients were obtained for the maximum, minimum, ground, and average temperatures in the pollen season. Negative correlation coefficients were stated for snow cover thickness and relative humidity. This means that the daily concentrations of alder pollen grains were strictly dependent on the daily temperatures. The temperature drop during the day entailed a decrease in the number of grains in the air. The presence of snow cover and high relative humidity had a negative impact on the presence of alder pollen grains in the air. Correlation coefficients, however, are low and explain the variability of diurnal concentration in a small degree depending on the weather conditions. This may be due to the delayed influence of individual weather elements on the release of *Alnus* pollen (Nowosad et al. 2018). In addition, the production and distribution of pollen grains is a complex process influenced by various factors, not only weather but also ecological, phenological, and genetic factors (Puc 2012; Nowosad 2016). Apart from the above-mentioned weather elements, the types of air masses and the direction of their inflow also impacted the alder pollen daily concentration (Tables 7, 8). According to Laaidi (2001), the type of atmospheric circulation (cyclonic or anticyclonic) may impact the concentration of pollen grains. According to the author, higher pollen grain concentrations are present in the case of anticyclonic situations. However, neither the type of atmospheric circulation nor the air mass inflow direction and type impacted the alder pollen grain concentration in Sosnowiec. The highest alder pollen grain concentrations were recorded during the inflow of warm air from the S + SWc (cyclonic situation from the south and south-west) direction and the lowest one in the case of the E + SEc (cyclonic situation from the east and south-east) and N + NEa (anticyclonic situation from the north and north-east) direction. The air mass type which particularly favoured the high daily alder pollen concentrations was warm maritime polar air (Fig. 4). The results of the analysis of variance for weather front type showed that the warm front is favourable to high concentrations of alder pollen grains, when the number of grains in the air is the greatest (Table 6).

In order to study the related meteorological factors and their impact on the alder pollen seasons, a linear regression model was built using regressive elimination (Table 9). However, the variation of the alder pollen grain number during the day was explained only in 5% by weather condition variability. It turned out that the strongest predictors were the average temperature and the relative humidity. Ranta and Satri (2007) studied alder pollen regression models as well; however, they obtained a very low determination coefficient, similarly as in the case of Sosnowiec. On the other hand, the multiple regression model proposed by Puc (2007) showed the minimum temperature and the average wind speed as the main weather elements that condition the presence of pollen in the air.

The alder pollen annual totals seem to be a little more complicated. In this case, their values are influenced not only by the weather factors of the current vegetation season, but also of the year preceding this season in the budding period (Sarvas 1972; Rodkiewicz 1973; Norris-Hill 1998; Rasmussen 2002; Ranta and Satri 2007; Stach et al. 2008). Norris-Hill (1998) reports that even the meteorological conditions from 2 years preceding the season may have an impact on the trees’ annual totals. The statistical analysis conducted for the alder pollen in Sosnowiec demonstrated that this taxon’s annual total is dependent on the maximum, minimum, and average temperature in the season as well as precipitation (Table 11). A negative correlation coefficient signifies that high temperatures in the pollen season have a negative impact on the annual total value. The situation with the meteorological conditions in the season preceding pollination is different. High positive correlation coefficients were obtained for thermal conditions and sunshine duration from the period from July to September (Table 12), while a negative correlation coefficient was stated in the case of this period’s precipitation. This means that the number of *Alnus* pollen grains was lower when high precipitation was recorded in the summer of the year preceding pollination. Similar results for alder annual totals were obtained in Lublin (Kaszewski et al. 2008).

Moreover, in the case of some tree species the occurrence of blooming abundance alteration, i.e. an endogenous cycle, which to a large extent is independent of the weather conditions, may be of great importance (Hallsdóttir 1999; Latorre 1999; Detandt and Nolard 2000; Jato et al. 2002; Lamontagne and Boutin 2007). This is manifested in the fact that years with high pollen production occur alternately with low production years.

In Reykjavik (Hallsdóttir 1999) and in Brussels (Detandt and Nolard 2000), a 3-year cycle was stated in the case of birch, which means that a year with high annual values is separated by 2 years with low values of the annual totals. In London (Emberlin et al. 1990), in Mar del Plata (Argentina) (Latorre 1999), in Copenhagen (Rasmussen 2002), in Gdansk (Latałowa et al. 2002), and in Santiago de Compostela (Spain) (Jato et al. 2002), a 2-year cycle was stated in the case of birch. From among other trees, oak demonstrated a 2-year cycle in London (Emberlin et al. 1990), Vienna, Leiden (Holland) and Brussels (Jäger et al. 1991) and even a 5-year one in Cardiff (United Kingdom) (Hyde 1952), and alder demonstrated a 2-year cycle in Draved Forest (Denmark) (Andersen 1980). However, the analysis of the annual totals of alder pollen grain in Sosnowiec did not demonstrate any existing cyclicality in the occurrence of years with high values of annual totals alternately with years of low pollen production. In the series under study, there are years with lower values of pollen grains separated by years with a high annual total, but not in regular time intervals.

The length of the alder pollen seasons was positively correlated with the relative humidity and with the average and maximum wind speed in the season, while negatively with the season temperatures (Table 13). According to Jabłoński and Szklanowska (1997), the pollination process takes place most intensely at low relative humidity and high air temperature. At warm sunny weather, the pollination period is short and its course is very regular. Low temperature and precipitation impact a prolongation of the blossoming period and disturb its regular course. In extreme cases, the pollen grain concentrations also drop due to underdevelopment of the thecae. This theory was confirmed to a large extent in Sosnowiec, at least when it comes to the temperatures. The alder pollinated shorter when the air temperature was high. Also, positive correlations were found with the average and maximum wind speed, which means that high wind speed prolongs the period in which pollen grains are present in the air. This was also been confirmed by the research conducted by other authors (Emberlin et al. 1997; Puc and Wolski 2002). High relative humidity also impacted the alder pollen season prolongation, whereas no significant dependence was found between the duration of the alder pollen seasons and the directions of air inflow and the air mass and weather front types.

The number of days with pollen grain concentration threshold values, which cause allergy symptoms, varied during the 21 years of observation. The most days with threshold values were stated in the years 2003, 2008, 2014, and 2016 (Table 1). These were concentrations which cause the first symptoms, i.e. 45 grains in 1 m^3^ of air and concentrations which cause symptoms in all persons hypersensitive to alder pollen allergens, i.e. 80 grains/m^3^ (Rapiejko et al. 2004). Thus, these were the seasons troublesome for all allergic to alder pollen allergens.

## Conclusions

Analysing the time of pollen season occurrence in the multiannual period studied, it was observed that the greatest differences concern the maximum concentration values and the pollen grain annual totals. This is proven by a high coefficient of variation and a high standard deviation value. The season characteristics which were the least varied in the years 1997–2017 were the end of the pollen season and the date of maximum daily concentration.

It was demonstrated that the differences in the subsequent pollen seasons are impacted by the weather conditions. The beginning of the pollen season was mainly conditioned by the thermal conditions in the months preceding pollination. Moreover, significant positive correlations were found with the temperatures occurring a long time before the season, i.e. from 210 to 180 days.

The value of the daily alder pollen concentration in Sosnowiec showed a statistically significant positive correlation with air temperature and sunshine duration, and a negative correlation with the thickness of the snow cover and relative humidity. The daily concentration also depended on the type of the weather front, the direction of air mass inflow, and the type of the inflowing air mass. The highest alder pollen grain concentrations were recorded during the inflow of warm air from the S + SWc direction and the lowest ones in the case of the E + Sec and N + NEa direction. The air mass type which particularly favoured the high daily alder pollen concentrations was warm maritime polar air. The results of the analysis of variance for weather front type showed that the warm front is favourable to high concentrations of alder pollen grains, when the number of grains in the air is the greatest.

The annual total (SPI) of the alder pollen grains also depended on the conditions present in the season as well as in the season preceding pollination. Statistically significant, negative correlation coefficients were found for the maximum, minimum, and average temperatures as well as precipitation in the season. However, in the case of the conditions from the preceding summer, high positive correlation coefficients were obtained for the temperatures and sunshine duration from the period from July to September, while a negative correlation coefficient was stated in the case of this period’s precipitation.

The duration of the alder pollen seasons was positively correlated with the relative humidity and with the average and maximum wind speed in the season, while negatively with the season temperatures.

The most troublesome for the allergy sufferers were the pollen seasons in the years 2003, 2008, 2014, and 2016, when the most days with threshold concentrations causing allergy symptoms were recorded.
